# Utilizing a steering committee to implement Health in All Policies in a rural, micro-urban community

**DOI:** 10.1093/heapro/daaf233

**Published:** 2026-01-31

**Authors:** Danika Lee Comey, Bridget L Hanson, Morgan E Neavill, Sarah P Church, Jennifer MacFarlane, Matthew Madsen

**Affiliations:** Mark and Robyn Jones College of Nursing, Montana State University, Bozeman, MT 59717, United States; Center for Health and Safety Culture, Montana State University, Bozeman, MT 59717, United States; Department of Psychology, Montana State University, Bozeman, MT 59717, United States; Department of Earth Sciences, Montana State University, Bozeman, MT 59717, United States; Western Transportation Institute, Montana State University, Bozeman, MT 59715, United States; Western Transportation Institute, Montana State University, Bozeman, MT 59715, United States

**Keywords:** Health in All Policies, social determinants of health, rural, steering committee

## Abstract

A multi-sector coalition in southwest Montana was established to bring a Health in All Policies (HiAP) approach to a rural and micro-urban community in southwest Montana. This project recruited local steering committee members from public health, government, transportation, social services, development, preservation, and education and organized them into work groups to design and implement rural-specific HiAP activities in their community. Workgroups were focused on ensuring community relevance and buy-in, developing and delivering curriculum, and data and evaluation. The steering committee met regularly during the project period; outcomes included a locally relevant project name and developing and delivering customized curriculum to multiple audiences. Post-implementation interviews were conducted with HiAP steering committee members (*n* = 7). Steering committee members report that participation in the HiAP project helped them better to understand social determinants of health, they believed in the mission of the steering committee, and they trusted the leadership of this collaborative work. A specific HiAP approach led by a multi-sector steering committee may be beneficial in helping to integrate HiAP in rural and micro-urban communities.

Contribution to Health Promotion

- This work contributes to health promotion by guiding rural communities on how they may implement a Health in All Policies approach in a rural or micro-urban community.
- This work helps to guide Health in All Policies in rural communities.
- This work examines lessons learned and implementation steps for those seeking to implement Health in All Policies in a rural community.

## Introduction

A multi-sector coalition in southwest Montana was established to bring a Health in All Policies (HiAP) approach to a rural and micro-urban community located in southwest Montana in the USA. HiAP is an international framework that integrates and leverages partnerships, collaboration, and community strengths to improve public health by influencing policies across non-medical sectors that impact health such as transportation, housing, urban planning, social cohesiveness, and education (Kahlmeier *et al*. 2010, Rigby and Hatch 2016, *Social Determinants of Health* 2022, Antin *et al*. 2024). There is a lack of research on how a HiAP curriculum and strategies could be adapted to fit the rural cultural context and ultimately implemented within rural and micro-urban communities. This paper seeks to communicate the utilization of a local multi-sectoral steering committee to adapt and implement HiAP in a rural and micro-urban community in the intermountain West; we provide recommendations to support implementation in other rural and micro-urban communities.

HiAP aims to improve community health, including analysis to understand how health is influenced by both the built and social environments in a community. By examining factors of the environment that contribute to health outcomes, the HiAP approach identifies “upstream drivers of health and social conditions” that must be considered at the policy-making level (Tonelli *et al*. 2020). It is paramount for decision makers and community leaders to examine how policy can impact health, and HiAP is a useful approach to understand and address how upstream factors contribute to poor health outcomes. HiAP is a highly important asset for both maximizing positive and minimizing negative impacts to population health (Khayatzadeh-Mahani *et al*. 2019).

The benefits of utilizing a HiAP approach within urban communities have been well documented (Corburn *et al*. 2014, Polsky *et al*. 2015, Hahn 2019). Most previous HiAP approaches have primarily focused on urban communities, whereas rural communities have been overlooked, especially in the USA (Leider *et al*. 2020). Although HiAP can be implemented at any level of government, most examples of implementation have been at the state level or within urban areas such as Richmond, California or Chicago, Illinois (Polsky *et al*. 2015, Hahn 2019). There is little existing research examining how a HiAP approach might be broadly implemented in a rural or micro-urban community where local policies are often preferred over state and federal policies. Expanding HiAP to rural and micro-urban communities in the USA is vital to addressing unique health disparities that impact those communities (Baldwin *et al*. 2021).

Barriers to accessing healthcare services have been documented in research among rural communities (Anderson *et al*. 2015, Polsky *et al*. 2015, Leider *et al*. 2020, Burch 2022). Rural populations often face greater barriers to accessing healthcare services when compared with their urban counterparts including the need to travel long distances to primary care, lower rates of insurance coverage, more complex health needs, and higher rates of poverty (Anderson *et al*. 2015, Leider *et al*. 2020). These factors have resulted in dire health inequities and disparities between urban and rural America (Anderson *et al*. 2015, Leider *et al*. 2020) including higher mortality rates for rural community members (Giannouchos *et al*. 2023). By implementing a HiAP approach, rural communities may see improvements in health and decreased inequities and disparities (Baldwin *et al*. 2021).

This paper contributes to reducing the knowledge gap related to implementing HiAP in rural and micro-urban communities by describing the utilization of a local multi-sectoral steering committee to guide the adaptation and initial implementation of a rural and micro-urban-specific HiAP in a rural and micro-urban community in the intermountain West. We describe the coalition’s process, implementation strategy, and results of evaluation activities as well as provide recommendations. Taken together, other rural and micro-urban communities can benefit from this experience to support HiAP work in their own communities.

### Context

There are many definitions of rural that are used across the literature. Definitions selected for this paper are supported from the Rural Health Information Hub (*Rural Health Information Hub* 2024). Rural areas are defined as areas where most of the population lives 60 miles or more from urbanized areas (areas with 50 000 or more population). Micro-urban is defined by the Office of Management and Budget, are labor market and statistical areas in the USA centered on an urban cluster (urban area) with a population of at least 10 000 but fewer than 50 000 people (*Rural Health Information Hub* 2024).

**Figure 1:**
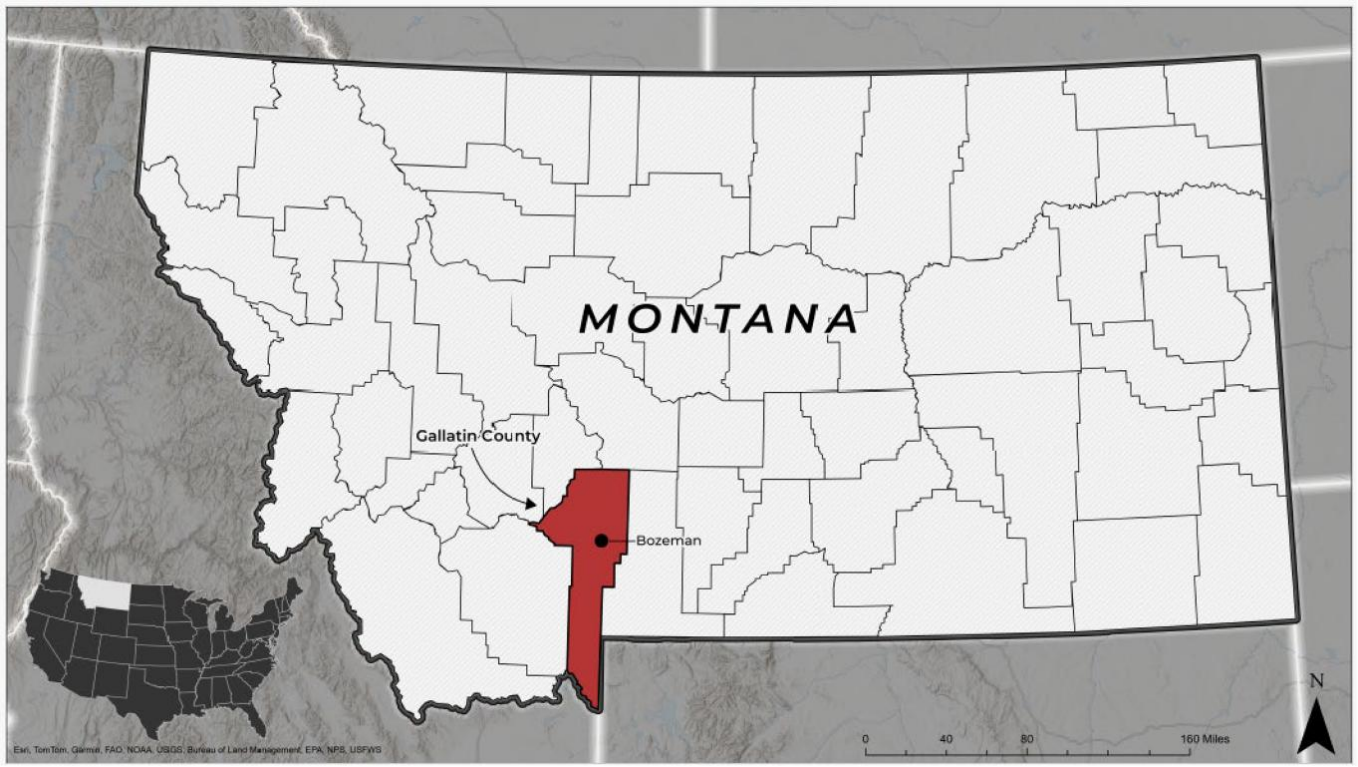
Context map. Map courtesy of the Geospatial Core Facility at Montana State University.

Gallatin County is in southwest Montana within the intermountain West and was selected as the site for the implementation of a rural HiAP approach. Gallatin County was selected as it is home to a public land-grant research university dedicated to focusing on rural health issues, training future rural health professionals, and located within a rural community (Fig. 1) (*About Montana State University* 2024). According to the Federal Office of Rural Health Policy, Gallatin County is a designated rural area (*USDA ERS - Rural Classifications* 2023, *Am I Rural?* 2025), although the main township in the county, Bozeman, recently achieved micro-urban status in 2020. The remainder of the county is classified as rural or frontier. Gallatin County stretches over 2205 square miles including large areas of federal lands containing geologic barriers such as mountains and rivers. The landscape causes a long commute with sometimes dangerous driving conditions for many rural residents to access work, healthcare, and social services in the micro-urban township of Bozeman.

### Process

A team of interdisciplinary researchers led by Montana State University’s (MSU) Western Transportation Institute began partnership with the Public Health Institute (PHI) in fall 2021 to discuss implementing a rural-specific HiAP approach in Gallatin County. PHI was contacted because of their expertise in prior HiAP implementation, and because of the need to support rural HiAP work. All work was reviewed and approved by the Institutional Review Board at Montana State University. With training and technical assistance received by PHI, the MSU research team recruited steering committee members. The research team intentionally recruited individuals and organizations to ensure representation by sectors that may not be involved in traditional public health partnerships such as transportation, community development, engineering, and the construction industry as collaboration between diverse sectors is an important aspect of HiAP (*Health in All Policies | Policy Performance and Evaluation | CDC* 2023). The research team relied heavily on personal connections and professional networking to recruit steering committee members (Williams 2002, Burbach *et al*. 2023). The research team contacted potential steering committee members to discuss the project, assess their interests, and invite them to participate. It was essential to the research team that the steering committee emphasize diversity, equity, and inclusion and attempted to ensure that professional organizations that work with historically underrepresented community members be included in this work. Ultimately, the steering committee consisted of MSU researchers along with representatives from the local health department, county-level government, and community organizations who together represented expertise in public health, government, transportation, development, and social sciences.

### Ethical approval

The interview protocol and procedures were reviewed and approved by the Montana State University Institutional Review Board. Interview participants were recruited via email and were provided a copy of the informed consent document prior to the interview.

The first steering committee meeting was held in December 2022 with 18 people in attendance. Steering committee members were academic researchers with expertise in public health and social science, students, developers, land preservationists, county-level elected officials, city government representatives, transportation officers, public health officials, and educators. At this first meeting, the research team and PHI provided context on HiAP and organized the steering committee into workgroups. During this initial steering committee meeting, it became apparent that steering committee members who worked outside of public health understandably lacked foundational knowledge important for successful HiAP implementation. Because a basic understanding of the Social Determinants of Health (SDoH) is essential for a HiAP approach, the second and third steering committee meetings included a presentation and in-depth discussion of SDoH and health equity in rural contexts, with local examples. This education was delivered by the project lead, with support from subject matter expert consultants and academic team members.

After learning about both HiAP and the SDoH, the steering committee decided to structure itself into working groups to implement HiAP in Gallatin County. Steering committee members volunteered to join a working group of their choice based on their interest, experience, and work sector. Three working groups were developed: the Community Relations Workgroup, the Curriculum Development Workgroup, and the Data and Evaluation Workgroup.

The Community Relations Workgroup was responsible for understanding and integrating the rural cultural context and marketing and promotion of the HiAP trainings, approach and for ensuring community buy-in. The Curriculum Development Workgroup was charged with designing and implementing a HiAP and SDoH community training that would be free and open to the public. The goal of the workgroup was to target the broad community, and the training would be open to any members of the public who were interested in this work. Intentional recruitment would target potentially interested sectors, but the trainings would be open to all. This workgroup would ensure that the developed curriculum was evidence-based, incorporated current literature, and was relatable to the community. The Data and Evaluation Workgroup was responsible for data collection related to program monitoring and evaluation. Each workgroup created their own action plan that included goals, priorities, strategies for implementation, and actions and activities for completion. Action plans were timebound and included specific target dates for strategy completion.

## Materials and methods

The Data and Evaluation Workgroup monitored the steering committee and workgroups. Process tracking included documentation of meeting dates, participation, and outcomes. Academic team members within each workgroup also documented barriers and facilitators they observed.

The Community Relations Workgroup conducted a convenience survey of community members (*n* = 41) for feedback on naming where participants could select all the names that they supported. Survey participants included engineers, planners, nurses, public health professionals, and students.

The primary data collection activity was steering committee interviews. These interviews were conducted with seven steering committee members in March 2024. The aim of these interviews was to understand how participating on the steering committee impacted members’ perspectives on and work related to HiAP as well as to understand what lessons can be learned and shared with other communities. An interview guide was developed by the Data and Evaluation Workgroup that consisted of six questions aimed at understanding participants’ experience on the steering committee: (1) their reason for participation, (2) if they would recommend colleagues to participate in a similar opportunity, (3) the best part of their experience, (4) what could have been better, (5) what their most significant change was as a result of participating, and (6) what influenced this change. Following these questions, participants were also given the opportunity to (7) provide additional comments or feedback.

The interview protocol and procedures were reviewed and approved by the Institutional Review Board at Montana State University. Interview participants were recruited via email and were provided a copy of the informed consent document prior to the interview. All interviews were conducted via Webex conferencing software. At the start of the interview, participants confirmed receipt of the consent form and agreed to being audio recorded during the interview. On average, interviews lasted approximately 20 minutes. All interviews were conducted by the same researcher (M.E.N.).

We used rapid qualitative analysis with a matrix technique to guide analysis and making meaning (St. George *et al*. 2023). Interviews were auto transcribed via Webex and the interviewer researcher reviewed the transcriptions for accuracy. The interviewer researcher then summarized each participant’s response to each question, resulting in a 7 (participants)×7 (questions) matrix and included key quotes. A second researcher (B.L.H.) reviewed the transcriptions and the summary matrix and suggested revisions as appropriate. The two researchers discussed and agreed on the final summary matrix. From that matrix, key themes were identified and described, with illustrative quotes selected.

## Results

 

#### Workgroup outcomes

As part of their role in ensuring community buy-in and marketing the HiAP curriculum and approach, the Community Relations Workgroup worked to determine the title of the project. This workgroup was composed of representatives from the sectors of public health, transportation, an elected government official, and community development. This was a challenging task, especially given the political environment of a rural community in Montana. Terms such as “health,” “equity,” and “inclusion” could isolate portions of the community and result in disengagement with more conservative segments of the population (Gollust *et al*. 2009). This was illustrated by one workgroup member who said they “Don’t want to even use the term health” to describe the training. Therefore, to select a name, the workgroup generated a list of potential names for the training and provided a blank/other option for individuals to provide their own idea for the training name. Respondents were able to select all the names they liked for the training. Results are listed in Table 1. Other naming options supplied by community members were the Health Communities Collaboration, Collaborating for Healthy Communities, and a Collaborative Approach to Building Livable Communities. The Community Relations Workgroup reviewed and discussed survey results and presented findings to the full steering committee. Following that presentation, the “Building Livable Communities” name was adopted for the project.

**Table 1:** Project naming.

Potential name	Percentage endorsing
Building Livable Communities	65.9% (27)
Health Community Collaboration	31.7% (13)
How to Design a Livable Community	22.0% (9)
Addressing SDoH through a HiAP Approach	9.8% (4)
Foundations of Wellbeing	9.8% (4)
Other	9.8% (4)

Responses total more than 100% as respondents could endorse more than one choice.

The Curriculum Development Workgroup was responsible for development and delivery of HiAP content to the larger community, and this workgroup identified three objectives to work toward. First was the development of a curriculum designed to train the community about HiAP and the SDoH. The second objective was to implement the curriculum in the community in various settings. The Curriculum Development Workgroup collaborated with other working groups to implement trainings in the community. The final objective was to create a HiAP Rural Toolkit that could be distributed to other rural, micro-urban, and frontier communities to help guide implementation of HiAP.

The Curriculum Development Workgroup created an interactive presentation for community members that first taught about the SDoH and then provided information on HiAP. The curriculum included evidence-based research on both the SDoH and HiAP. The curriculum included specific regional examples of the SDoH as well as facilitated conversations with community members about how to make positive change in the community and how to address SDoH. A key aspect of the curriculum was to break the workshop participants into small groups. The intended audience included public health and the sectors critical for HiAP that public health in Gallatin County does not often collaborate with, such as engineers, developers, and elected officials. The workgroup envisioned that diverse groups could receive training together and that by working in small groups throughout the curriculum, community members from different organizations, departments, and institutes had an opportunity to network, engage, and build relationships.

The Curriculum Development Workgroup worked intentionally to include specific examples and images from the Gallatin County community, including images and experiences that the audience could relate to. It was also crucial to the workgroup that the curriculum could be delivered to community members from diverse sectors with trusted facilitators. Professionals from the workgroup who were trained in curriculum facilitation included representatives from public health, transportation, city planning, county planning, and local university graduate students; these workgroup members were willing to deliver initial training and build capacity of others to deliver future training.

**Figure 2:**
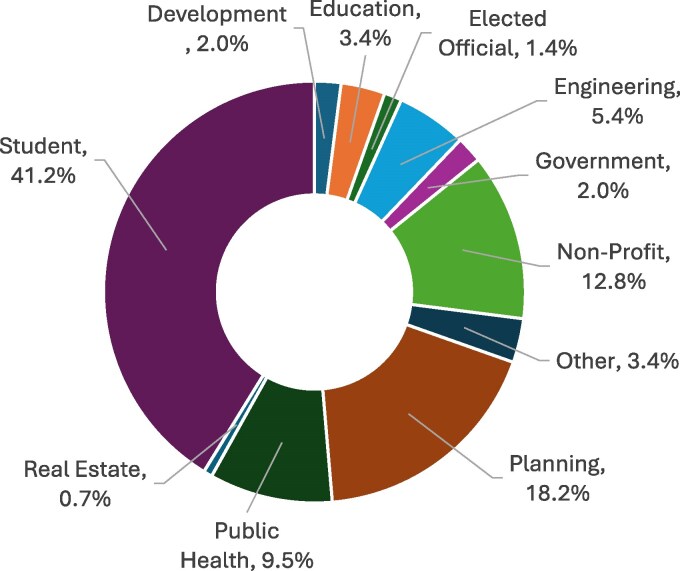
Training attendee sectors.

The curriculum was first pilot tested at the 2023 Montana Association of Planners conference in Butte, MT in September 2023. A second pilot test was conducted with the Gallatin City-County Health Department staff later in fall 2023. Steering committee members attended both pilot studies and took notes on the curriculum delivery and feedback from the participants. After the pilot studies were conducted, the Curriculum Development Workgroup worked with the steering committee to update the curriculum. The updated curriculum was then presented to the public as free and open community events. These events were intended for the broad range of professional stakeholders critical for implementation of HiAP; intentional promotion was conducted in order to recruit attendees from identified sectors that are often missing or overlooked. For example, steering committee members worked personally to invite engineers and elected officials and partners promoted the opportunity via professional newsletters and networks. Additionally, trainings were promoted broadly to the community in the local newspaper and other avenues (e.g. newsletters, social media) and members of the general public were welcome to attend. The curriculum was structured as a presentation with slides and included discussion questions; sessions lasted 90 min. The community sessions of the curriculum were facilitated at different times of day (e.g. evening as well as daytime sessions) to provide training that could work with different schedules. Training courses provided a free meal and free childcare for attendees. Additionally, abridged HiAP trainings (60 min) were delivered at the local university as guest lectures in geography, community health, and engineering courses and to the local city-county board of health. Delivery to students in diverse disciplines allowed the curriculum to reach future professionals. Ultimately, training was delivered via 2 pilots and 7 presentations to 158 individuals in 2023 and 2024. Sectors represented at these trainings are shown in Fig. 2.

The Curriculum Development Workgroup collaborated to create a short, informative toolkit designed to guide other rural and frontier communities in implementing a HiAP curriculum. The toolkit, published by Montana State University Western Transportation Institute, outlines and describes the workgroup’s process in ten clear steps, providing a structured approach that other rural communities can adapt to fit their unique needs. By offering practical guidance and insights, the toolkit serves as a valuable resource for fostering cross-sector collaboration and integrating health considerations into local decision-making.

**Figure 3:**
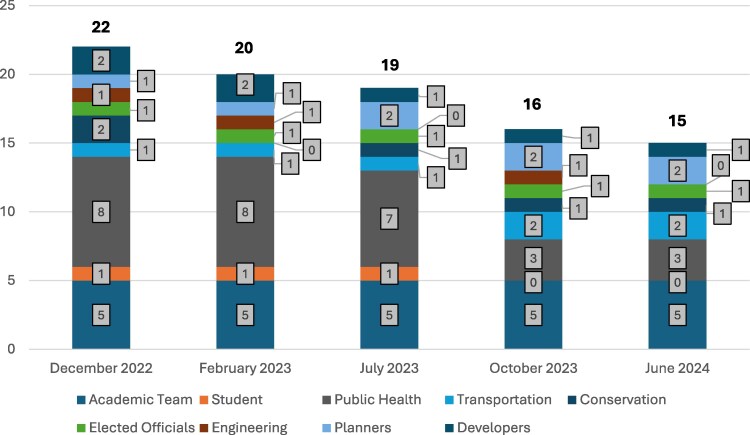
Steering committee meeting attendance by sector.

The Data and Evaluation Workgroup was responsible for programmatic evaluation of the HiAP approach. The workgroup created a logic model early in the implementation process to guide the steering committee and workgroups; the logic model was presented to the steering committee and revised based on feedback. Participation tracking revealed decreased participation over time but representation by diverse sectors throughout the early implementation period (Fig. 3). Across sectors, seniority of the steering committee members also varied somewhat. As the largest sector, public health included job titles such as intern, program associate, and director. Similarly, the academic team included faculty, research staff, and students. Other sectors were represented on the steering committee by fewer individuals and those people tended to have senior roles, with job titles such as senior planner, senior engineer, associate director (within the conservation sector), president (within the development sector), and county commissioner.

### Interview findings

#### Reasons for participation

Participants primarily indicated willingness to participate on the steering committee because they were asked by someone whom they had an established, positive working relationship. Participants described the project leader as credible, respected, and respectful of others. Respondents also reported they desired to participate because they believed in the mission of incorporating health into all aspects of the environment. One participant said this is: “important for not only individual health but community wellbeing and building social cohesion” (p. 4). They were especially excited for an opportunity to work on this mission via collaboration with other community members.

#### Recommending colleagues

Most participants reported that they would “absolutely” or “definitely” recommend colleagues to participate in a similar opportunity to serve on a HiAP committee in their own community. Several participants said they would recommend a colleague to participate with certain caveats, such as if it fit within the individual’s community context and they could commit to the amount of time necessary to serve on the committee: “It depends a little bit on the context of the community. But generally speaking, I think it’s worthwhile to be involved even if the project doesn’t perfectly fit the context that you work in… there's still lessons to be learned either personally, or, you know, that you can apply professionally in other situations. So, there's value in it and other ways outside of your specific job that you have currently in this moment.” (p. 1). Another participant described the importance of job fit in a different way, reflecting that some positions may be better suited for success and satisfaction with the steering committee work, saying “I think it would depend on what they were doing and their broader vision of how their work fits in. Even though we’ve tried to put it in front of a lot of different sectors, I don’t know that it’s been 100% successful. Not necessarily because of the efforts, just because I think not everybody gets it, and not everybody may get it.” (p. 7).

One participant recommended others participate due to the powerful impact multi-disciplinary efforts can have on one’s community: “These efforts, what’s exciting and equally very hard about them, is that they are multi-disciplinary, and they cross over so many sectors, in our local governments, and our community. You could not do it just by government policies, you could not achieve what you would ultimately want to just with a few good private sector folks trying to move the needle. Right? So, it’s that public-private partnership and across disciplines. Looking at health and wellness as well as education and our built environment, again. So, like all of those pieces really working together.” (p. 4).

#### Experience highlights & areas for improvement

One of the most common themes mentioned throughout the interviews was the importance and benefit of collaboration with others in the community. Several participants specifically highlighted that they enjoyed the opportunity to collaborate with researchers from the local university. One participant described that they appreciated “the opportunity to interface with MSU folks in particular, it's been really cool because of the scientific or evaluation-based approach that we've been taking.” (p. 6).

Logistically, participants appreciated having efficient and well-organized meetings along with project leaders that respected people’s time and were transparent on expectations. Participants described meeting facilitators that were willing to discuss anything and were never thrown off guard by questions because they were well-prepared and willing to engage in discussions even when they did not have answers; interview participants noted that this skill and approach supported successful steering committee meetings. Participants were satisfied with the structure of meeting and working as subcommittees, then having check-ins as a whole group.

One participant indicated that the meetings could be long and slow-paced. Other participants described collaboration and networking at meetings as the best part of their experience, including things like opportunities at meetings to provide other updates from their organizations or agencies, which all take time. When describing what could have been better logistically, a couple participants mentioned retention, referring to some rotation in steering committee members, with members dropping off and new members joining. However other participants described retention as a strength, with statements like “people weren’t falling off.” (p. 4) because the commitment was meaningful and worthwhile.

Regarding HiAP content, participants found value in learning more about SDoH. Several participants indicated the importance of making sure the content is careful to include applied examples: “I don’t mean this as a criticism, because I think to some degree, this is important, but [the SDoH content] may have been a little academic at times. And so, we are living in a very applied world, at least for me. Like a researcher, a professor, or a graduate student serving on the committee, super comfortable, right? Like they’re kind of enmeshed in that, but I’ve been out of school a long time.” (p. 4). Another participant indicated an area for improvement in connecting the content to applications: “I’ve tried to start incorporating some of the things I’ve learned into my day-to-day job, but it’s still challenging because, you know, I understand the concepts, and I understand what I do for work, but I’m still figuring out how to connect those two things.” (p. 1).

Some steering committee members also delivered the curriculum in trainings to their colleagues and to community members. When discussing the development of training content and its delivery, one participant discussed how more prep time, practice presenting, and more group development could have been helpful, but indicated that these skills were developed with practice and time: “Considering now that we have done a couple additional trainings and now we’re going to go through another round of it, and it sounds like it’s just getting more and more tight, I don’t have a lot of criticism on it. Just something that takes time.” (p. 3).

Overall, many participants reported that they appreciated the clear aims and directions for the steering committee and how the work felt purposeful: “I felt like the agendas and how the project was structured, just really, when we were together, we were working on something, and it was clear what we were accomplishing.” (p. 4). However, one participant reported previous experience with similar initiatives that they thought did not clearly result in meaningful community change and conveyed concern this will have the same outcome, saying “I see a whole bunch of these types of things go through and most of them don't add up to result in anything.” (p. 2).

#### Most significant change

When asked to think about and describe the most significant change they have experienced as a result of participating on the steering committee, participants frequently discussed their increased knowledge on SDoH: “I can actually tell you more about the SDoH and why health really needs to be incorporated more into policy making.” (p. 3). This increased knowledge, along with practice, led several participants to have increased confidence in their ability to present on this topic. Several people also mentioned having a better understanding of what others do in the community, seeing common threads and overlap, and thinking about the need to break down silos between agencies and sectors: “I think more mindfully about what collaboration means and how we can use that to better impact our services to provide better outcomes for people in the community.” (p.5). Participating has given them a better understanding of how their work impacts health overall.

Participants reported beginning to notice how HiAP concepts are being implemented in other community agencies and organizations: “What I’ve appreciated seeing now, I’m doing some project for the hospital, for instance, and I’ve seen that they are now referencing HiAP in a couple things that they’re doing” (p. 7). Participants discussed beginning to think about HiAP in their own work as well, ranging from staffing considerations to conversations with colleagues: “I think what this project has allowed me to do is speak more comfortably toward peers and partners about the importance of collaboration. Really bringing that discussion up with coworkers has really put that in their minds too and really gotten them interested in the discussions about it.” (p. 5). The most common theme participants discussed when asked what influenced their change, was having exposure to what others are doing in their work and community and being able to learn from one another.

One participant discussed how as a result of participating, they now are better at finding connections between things they would previously assume to be unrelated, and can now take a more holistic approach in their work. They also mentioned a deeper appreciation for evaluation and understanding impact: “I think I have a little bit of a keener eye toward evaluation and the research component of this, and the way that we have been working with folks at MSU, I’m assuming this is part of just like the grant funding that kind of built in this evaluation, but I just think it's good practice whether or not, you need to do it. Like, okay, what are our goals that are we trying to achieve? …. I think a lot of us in the non-profit sector are really good at coming up with ideas to solve problems, and then we don’t take the time to see if we actually did. We just kind of take people’s word for it and move on. I think the evaluation piece has been a good lens for me to have since we started.” (p. 6).

#### Recommendations

While interview participants were not specifically asked about their recommendations for other steering committees doing this work, some provided insights that may be helpful. Participants encouraged other communities to engage in similar efforts to promote HiAP and hope to see it continue in their community. A recommendation is to make sure that key people are receiving this message: “I'd love to see it replicated in other places. I'd love to see it kind of continue on here because I think there's a lot of people that need to hear this message that haven’t. I think we got the low hanging fruit, the people that were easiest to get to, now there’s a lot of really key influential people that either don’t want to hear this, or don’t have time to hear it and we need to get to them.” (p. 6). Several participants emphasized the need for there to be a seat at the table for developers, private sector representatives, and others who will be implementing this work. This ensures that they are included in the discussions from the beginning and increases collaborative efforts and potentially willingness to implement these concepts. One participant stated: “I thought that the selection of the steering committee was very thoughtful and very inclusive. I know it's hard, but maybe if I were to repeat it, trying to maybe recruit another private sector representative or two. You know, just because it's so varied in the community, I think that would have been helpful for the process.” (p. 4).

Participants acknowledged the need for ongoing work and sustained effort to make lasting change in the community. One participant suggested a course or continued HiAP training on what the concepts look like applied: “I just hope that we can continue this project and kind of evolve it to increased action. I mean, I think that we have laid a great foundation for these discussions in the community. I think, like I said, there's a lot of excitement around the discussions amongst people on the committee and people that have taken the training. I think that at some point, there will be, the need for next steps, kind of once you've gone through this training, how can you help in different ways? I think that will be, as we do more presentations, I think that will become an increasing need.” (p. 5).

## Discussion

The Building Livable Communities steering committee was successful in establishing a diverse steering committee, developing a culturally appropriate HiAP curriculum, implementing a HiAP approach, and leading this work in a rural community. This research focuses on how to successfully implement a HiAP framework in a rural community. Previous literature and research have examined the success that urban and suburban communities can have when implementing a HiAP approach in their community to address the SDoH (Polsky *et al*. 2015, Hahn 2019, Baldwin *et al*. 2021). However, less was known about the feasibility of a culturally appropriate community education and if the HiAP approach could be tailored to fit the unique needs of rural and frontier communities, especially in the USA. We documented an experience utilizing a diverse steering committee to successfully share HiAP and SDoH education. Steering committee members reported increased knowledge in these areas as well as enhanced collaborations with other community members and professionals from diverse sectors and fields toward shared goals of improving health.

HiAP philosophy stresses the importance of collaborating with diverse partners and stakeholders (*Health in All Policies | Policy Performance and Evaluation | CDC* 2023). The steering committee was intentional about the selection of community partners to work with to adapt curriculum and pilot a HiAP approach. It was important to include representatives from a variety of sectors and especially imperative to ensure the collaboration of organizations outside the medical and public health fields.

Moreover, the research team specifically worked with partners outside the health sector to discuss what terminology should be used in the HiAP approach. Although a diverse steering committee is important in HiAP work, it may take additional efforts to engage non-health sectors where their connection to HiAP work may not be as clear. Words such as “health” and “equity” can be seen as political and can alienate community members; therefore, HiAP efforts must be mindful of language to include all community members (*Racial Equity* 2024). Despite potential backlash for including these topics, the steering committee proceeded with curriculum development and dissemination as addressing SDoH is critical for improving community health and decreasing health disparities (Marmot 2005, Gollust *et al*. 2009). Diverse partnerships were beneficial in choosing common language that supports community collaboration and participation in educational events.

When considering the implementation of a HiAP approach in rural communities, it is important to be aware that many see community health work as political (Borrell *et al*. 2007). This therefore requires collaboration with individuals and community partners in a non-threatening environment to deescalate tensions and to collaborate toward the common goal of making communities livable for all. In our work, steering committee members appreciated the structure of meetings that were efficient and well-organized and yet also provided members the opportunity to share their own updates and explore additional collaborations.

The committee worked through numerous decisions and tensions as they developed this program. For example, the Community Relations workgroup was tasked with finding a nonpolitical, inclusive name for the community training. The workgroup discussed at length whether to use the term health to describe the training. The members of the workgroup were fresh from the COVID-19 pandemic and were hyperaware that many community members may be dissuaded from participating in work that revolved around health. As such, the team decided to ask the community for feedback on the most affirming term for this training. When determining the best name for this work, the group also wanted to emphasize that this work was a county-wide effort, not just a project in the micro-urban environment in the county, so it was important that it was not named after the city.

A common challenge in prevention work, including HiAP, is that the outcomes will not be visible for years or decades to come. By working to address conditions that lead to poorer health and health inequities, it will take years or decades for the impacts of this intervention to be seen in the community. However, there is national, evidenced-based research that indicates that working on the underlying conditions that influence health outcomes will have positive impacts on building healthy, equitable, and livable communities. While steering committee members initially had different levels of understanding and engagement with public health and SDoH, they shared a value related to wanting good health for their community. After participation in the steering committee, interview findings reflected that members had shared appreciation for addressing SDoH and supporting HiAP in their community. Although additional funding has not been secured to formally continue this project, engaged steering committee members have continued this work in their cross sectoral roles across the community to promote HiAP and to continue conversations about the SDoH. This paper presents our experience with a steering committee to implement HiAP in a rural community, including findings about the implementation process, workgroup outcomes, and findings from interviews with steering committee members. One major limitation is that this work was done in one rural and semi-urban geographic area, and results may not generalize to other communities. Nonetheless, other communities and individuals interested in conducting similar work may benefit from recommendations, which include engaging diverse and key community representatives for a steering committee, utilizing their time and talents intentionally and efficiently, ensuring respected and effective leadership for the steering committee and HiAP effort, and adapting educational materials and resources careful to match community context. The field would benefit from future research to further examine HiAP in rural settings and the role of steering committees and other facilitators, particularly with regard to supporting long-term and lasting change across professional sectors and throughout communities.

## Conclusion

A collaborative academic-community partnership worked together to successfully bring a HiAP approach to a rural community in southwest Montana. Through the collaborative process a steering committee came together to create and implement a curriculum that educated community members about the SDoH, HiAP, and provided opportunities for networking and cross sectoral collaboration. Prior to this work, little research existed on how a HiAP approach could be adapted to meet the cultural needs of a rural community.

Rural communities are often unnoticed and overlooked. There are significant health inequities between rural and urban populations and the adaptation and implementation of a rural HiAP approach may be one way to address and dismantle health disparities. Now is the time for this work. Current conditions in some rural communities exist for successful HiAP education. This research demonstrates that community organizations and individuals are passionate about community needs and committed to engaging in efforts to address challenging issues. The problems facing rural communities are urgent, and community organizations and individuals want to work together to develop solutions using a HiAP approach.

## Data Availability

The data supporting the findings of this study are available from the corresponding author upon reasonable request.
